# SIRT1 pathway in Parkinson’s disease: a faraway snapshot but so close

**DOI:** 10.1007/s10787-022-01125-5

**Published:** 2022-12-29

**Authors:** Gaber El-Saber Batiha, Hayder M. Al-kuraishy, Ali I. Al-Gareeb, Engy Elekhnawy

**Affiliations:** 1 Department of Pharmacology and Therapeutics, Faculty of Veterinary Medicine, Damanhour University, Damanhour, 22511 AlBeheira Egypt; 2 Department of Pharmacology, Toxicology and Medicine, College of Medicine, Al-Mustansiriyah University, Baghdad, 14132 Iraq; 3 Pharmaceutical Microbiology Department, Faculty of Pharmacy, Tanta University, Tanta, 31527 Egypt

**Keywords:** Silent information regulator, Parkinson’s disease, Oxidative stress, Inflammatory disorders, Mitochondrial dysfunction, Apoptosis

## Abstract

Silent information regulator (SIRT) has distinctive enzymatic activities and physiological functions to control cell-cycle progression, gene expression, and DNA stability by targeting histone and non-histone proteins. SIRT1 enhances synaptic formation and synaptic activity, and therefore, can reduce the progression of various degenerative brain diseases including Parkinson’s disease (PD). SIRT1 activity is decreased by aging with a subsequent increased risk for the development of degenerative brain diseases. Inhibition of SIRT1 promotes inflammatory reactions since SIRT1 inhibits transcription of nuclear factor kappa B (NF-κB) which also inhibits SIRT1 activation via activation of microRNA and miR-34a which reduce NAD synthesis. SIRT1 is highly expressed in microglia as well as neurons, and has antioxidant and anti-inflammatory effects. Therefore, this review aimed to find the possible role of SIRT1 in PD neuropathology. SIRT1 has neuroprotective effects; therefore, downregulation of SIRT1 during aging promotes p53 expression and may increase the vulnerability of neuronal cell deaths. PD neuropathology is linked with the sequence of inflammatory changes and the release of pro-inflammatory cytokines due to the activation of inflammatory signaling pathways. In addition, oxidative stress, inflammatory disorders, mitochondrial dysfunction, and apoptosis contribute mutually to PD neuropathology. Thus, SIRT1 and SIRT1 activators play a crucial role in the mitigation of PD neuropathology through the amelioration of oxidative stress, inflammatory disorders, mitochondrial dysfunction, apoptosis, and inflammatory signaling pathways.

## Introduction

Silent information regulator (SIRT) comprised various class III histone deacetylases; seven types of SIRT are present SIRT1–SIRT7 which have distinctive enzymatic activities, physiological functions, and sub-cellular localization. SIRT1 was primarily recognized as a nuclear protein involved in tumor progression, neuronal differentiation, and apoptosis. Later on, SIRT1 had been observed to control cell-cycle progression, gene expression, and DNA stability by targeting histone and non-histone proteins.

SIRT1 is a nuclear protein sequestered in the cytoplasm according to the cell response to stress (Alam et al. 2021). SIRT1 is involved in different physiological functions including DNA repair, genomic stability, apoptosis, antioxidant effect, and enhancement of mitochondrial biogenesis (Yanagisawa et al. 2018; Alam et al. 2021). SIRT1 promotes the extension of the life span through the downregulation of the aging process (Chen et al. 2020). In addition, SIRT1 enhances synaptic formation and synaptic activity (Peng et al. 2022), therefore can reduce the progression of various degenerative brain diseases like Alzheimer’s disease (AD) and Parkinson’s disease (PD) (Wang et al. 2010). It has been shown that SIRT1 activity is decreased by aging with a subsequent increased risk for the development of degenerative brain diseases (Chen et al. 2020).

**Fig. 1:**
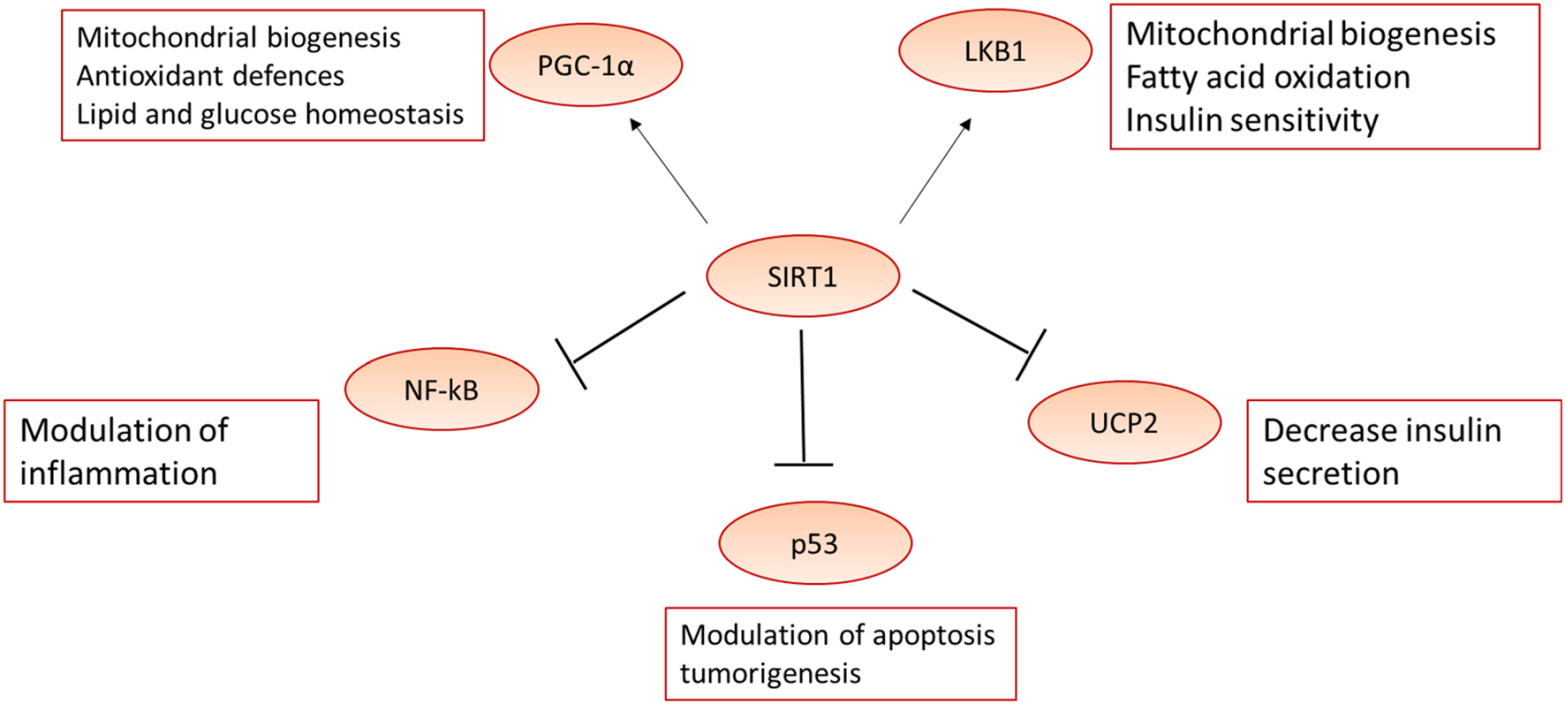
Role of SIRT1 in cellular functions. Adenosine monophosphate protein kinase (AMPK) is activated by oxidative stress leading to the enhancement of mitochondrial biogenesis, fatty acid oxidation, and improvement of insulin sensitivity. AMPK increases the NADH ratio leading to activation of SIRT1 which via expression of liver kinase B (LKB1) stimulates AMPK expression. Activated SIRT1 stimulates peroxisome proliferator-activated receptor gamma co-activator 1 alpha (PGC-1α) leading to improvement of mitochondrial biogenesis, antioxidant effects, and regulation of glucose as well as lipid homeostasis. As well, activation of SIRT1 inhibits expression of p53 and transcription of nuclear factor kappa B (NF-κB) with subsequent inhibition of apoptosis and inflammation, respectively

SIRT1 is a NAD-dependent deacetylase protein encoded by the SIRT1 gene. SIRT1 acts as an intracellular regulatory protein that regulates epigenetic genes (Hu et al. 2019). SIRT1 affects the transcription of different regulatory proteins involved in metabolic effects (Hu et al. 2019). SIRT1 activates peroxisome proliferator-activated receptor gamma co-activator 1 alpha (PGC-1α) and estrogen-related receptor alpha (ERRα) which is also known as a nuclear receptor 1 (Wan et al. 2022). Moreover, SIRT1 interacts with various molecular pathways including hairy enhancer of spilt related with YRPW motif protein 2 (HEY2), autoimmune regulator (AIRE), miR-132, and poly ADP-ribose polymerase 1 (PARP-1) (Wahab et al. 2021). As well, SIRT1 deactivates the p53 protein preventing apoptosis and cell deaths (Khanahmadi et al. 2020). In addition, SIRT1 activates autophagy by inhibiting the acetylation process which is necessary for the induction of autophagy (Li et al. 2019). SIRT1 regulates the immune response via stimulation of T-helper 17 cells which are involved in the induction and development of autoimmunity (Jia et al. 2018). SIRT1 is closely related to addiction pathogenesis through interaction with histone deacetylase 1 (HDAC1) and AP-1 promoter in the dopaminergic neurons (Engel et al. 2016). Therefore, SIRT1 affects different cellular processes involved in DNA damage and oxidative stress (Kwon et al. 2019) (Fig. 1).

There are different types of SIRT1 activators including lamin A protein, metformin, resveratrol, SRT-1720, and methylene blue (Hubbard and Sinclair 2014). Resveratrol and SRT-1720 directly activate SIRT1 or indirectly through the activation of AMP-activated protein kinase (AMPK) which is the cofactor for SIRT1 activity (Liang et al. 2020). Increasing NAD level plays a role in the activation of the SIRT1 pathway (Liang et al. 2020).

Furthermore, inhibition of SIRT1 promotes inflammatory reactions since SIRT1 inhibits transcription of nuclear factor kappa B (NF-κB) which also inhibits SIRT1 activation via activation of microRNA and miR-34a which reduce NAD synthesis (Qian et al. 2018). Both PARP-1 and SIRT1 required NAD for their activations (Qian et al. 2018). PARP-1 is necessary for DNA repair; thus, high DNA injury induces a reduction in NAD level and then in SIRT1 activity (Bai et al. 2011).

Since SIRT1 has antioxidant, anti-inflammatory, and genomic stability effects, and it is highly expressed in microglia and neurons in the human brain (Cho et al. 2015), this review aimed to find the possible role of SIRT1 PD neuropathology.

## Parkinson’s disease (PD) overview

PD is a common chronic degenerative brain motor disorder after Alzheimer’s disease (AD) (Blauwendraat et al. 2020). PD is a progressive neurological disease that develops due to injury of the dopaminergic neurons (DNs) in the substantia nigra (SN) (Bloem et al. 2021; Armstrong and Okun 2020). PD neuropathology is characterized by the deposition of α-synuclein in the SN with the formation of Lewy bodies (Lang et al. 2022). PD is characterized by the formation of Lewy bodies which consist of aggregated α-synuclein, ubiquitin, and heat shock protein (HSP) (Yang et al. 2020; Church 2021). Lewy bodies are not limited to the brain but are also present in other organs including the spinal cord, autonomic nervous system (ANS) (Carapellotti et al. 2020), intestines, and pancreas. α-Synuclein may be initially formed outside the CNS and then transmitted to the CNS via cranial nerves like prions. Besides, α-synuclein aggregation is not limited to the SN but also affects many brain regions including aggregation of α-synuclein (Chen et al. 2019). Deposition of α-synuclein is started at first in the ANS, mostly in the dorsal motor nucleus of cranial nerves, and then spread to the other brain regions (Norcliffe-Kaufmann 2019). PD affects 1% of the general population over the age of 60. PD was initially recognized in 1817 by James Parkinson who describes shaking palsy (Bloem et al. 2021). PD is characterized by motor symptoms like rigidity, resting tremor, and bradykinesia (Lang et al. 2022) and non-motor symptoms like apathy, depression, anxiety, cognitive dysfunction, and neuropsychiatric as well as sleep disturbances (Yang et al. 2020). Notoriously, non-motor symptoms are developed by many years before starting motor symptoms. Markedly, non-motor symptoms are mounting before degeneration DNs in the SN (Durcan et al. 2019). Following the development of motor symptoms, cognitive dysfunctions progress due to the involvement of the atemporal cortex (Kalia 2018). Different factors are involved in the pathogenesis of PD including old age, genetic, epigenetic, and environmental factors causing increasing deposition of α-synuclein and formation of Lewy bodies. These changes induce microgliosis, mitochondrial dysfunction, oxidative stress, and inflammation with further dopaminergic neuronal loss in the SN and the development of motor symptoms in PD. Therefore, PD neuropathology is intricate and connected to different factors (Yang et al. 2019).

## Neuroprotective effects of SIRT1

SIRT1 exerts a neuroprotective effect through the regulation of the expression of transcription factors including forkhead box O3 (FOXO3A), p53, E2F transcription factor 1 (E2F1), and C-terminal binding protein (CtBP) (Lu and Wang 2017). SIRT1 plays a critical role in neuronal differentiation, memory, and learning functions as well as cognitive function and neurogenesis (Cao et al. 2017). SIRT1 is highly expressed in the CNS, mainly the brain, spinal cord, and ganglions during embryonic life (Quiñones et al. 2021). However, in adult life, SIRT1 is expressed mainly in the hippocampus and cerebral cortex (Sasaki and Kitamura 2010). This expression is inversely correlated with aging (Chen et al. 2020). Different preclinical studies revealed that SIRT1 has neuroprotective effects through modulation of the fate precursor cells, inhibition of p53, and attenuation of axonal degeneration during cerebral ischemic/reperfusion injury (Bonfili et al. 2018; Ma et al. 2020). Therefore, downregulation of SIRT1 during aging promotes p53 expression and may increase the vulnerability of neuronal cell deaths following ischemic stroke.

Moreover, SIRT1 reduces neuronal injury by inhibiting the expression of inducible nitric oxide synthase (iNOS) which contributes to the aggravation of neuronal injury via the activation of glutamate neurotoxicity (Zhang et al. 2021). Neuroinflammation is linked with different neurological disorders including traumatic brain injury, ischemic stroke, meningitis, PD, and AD. SIRT1 reduces the expression of inflammatory signaling pathways and the release of pro-inflammatory cytokines (Jiao and Gong 2020). Similarly, SIRT1 attenuates the development and progression of neuroinflammation by inhibiting expression of NF-κB, cyclooxygenase-2 (COX2), and release of pro-inflammatory cytokines like IL-1β and tumor necrosis alpha (TNF-α) (Jiao and Gong 2020). These inflammatory cytokines act in a loop to activate COX2 with further release of pro-inflammatory cytokines and induction of BBB disruption and vascular injury.

Furthermore, apoptosis plays a crucial role in neuronal damage following brain ischemic reperfusion injury (Hollville et al. 2019). The apoptotic pathway is activated following focal brain ischemia leading to progressive neuronal injury via the activation of caspase-3 (Kuzu et al. 2018). In addition, microRNA is high in the CNS that plays a role in the regulation of growth and differentiation of cerebral neurons via targeting of various genes involved in neuronal growth (Ma et al. 2019). SIRT1 activates microRNA expression leading to decreasing in infract squeals following brain ischemic injury (Xu et al. 2021). These findings highlighted the molecular effects of SIRT1 through modulation of DNA repair, autophagy, energy metabolism, anti-inflammatory, and antioxidant effects as well as improvement of cerebral blood flow (CBF) (Ma et al. 2019; Xu et al. 2021). Therefore, the neuroprotective effect of SIRT1 is mainly mediated by activation of neurogenesis, synaptic plasticity, improvement of mitochondrial function, regulation of BBB permeability with inhibition of oxidative stress, and accumulation of α-synuclein and tau proteins (Zhang et al. 2018, 2012; Abo-Sleiman et al. 2006; Yang et al. 2022).

## SIRT1 in PD

SIRT1 is a highly preserved NAD-dependent deacetylase involved in protection against different degenerative brain diseases (Chen et al. 2021). Genetic variation in SIRT1 is linked with the development of sporadic PD as documented in preclinical studies. As well, genetic mutations in PD had been reported to alter the expression of the SIRT1 promoter with subsequent reduction of the neuroprotective role of SIRT1. Transgenic mice with over-expression of SIRT1 are resistant to MPTP-induced PD through inhibition expression of pro-inflammatory cytokines and microglia/astrocytes activation (Maszlag-Török et al. 2021). SIRT1 through interaction with heat shock protein A4 (HSPA4) represses the expression of pro-inflammatory cytokines. Therefore, SIRT1/HSPA4 complex could be a therapeutic target to mitigate neuroinflammation in PD. Chen and colleagues revealed that SIRT1 protects DNs against MPTP-induced injury through the improvement of mitochondrial function, reduction of the apoptotic process, and activation of PGC-1α (Zhang et al. 2018). In this state, the experimental study of the PD model showed that mutated SIRT1 failed to protect DNs (Donmez et al. 2012; Chen et al. 2019; Zhu et al. 2021). Thus, SIRT1-mediated mitochondrial biogenesis could be a possible mechanistic way for the neuroprotective effect of SIRT1 against PD neuropathology. It has been shown that SIRT1 expression was reduced in toxin-induced and genetically modified PD models (Ubaid et al. 2022; Mohamad et al. 2022; Pallas et al. 2008; Guo et al. 2016). In vitro study demonstrated that SIRT1 attenuates degeneration of DNs by increasing expression of FOXO3a and hypoxia-inducible factor 1 alpha (HIF-1α) with inhibition of oxidative stress (Rodriguez et al. 2018). In addition, the neuroprotective effect of indol-3-carbinol against PD is mediated by SIRT1 (Singh et al. 2017). These preclinical studies indicated that dysfunctional SIRT1 is associated with PD pathogenesis. A case–control study illustrated that SIRT1 mRNA level was reduced in the peripheral blood of PD patients (Maszlag-Török et al. 2021). An extended study involving PD with dementia and healthy controls illustrated that SIRT1 expression significantly differed between PD patients with dementia compared to the healthy controls (Zhu et al. 2021). SIRT1 expression was reduced in the frontal and temporal lobes of PD patients with dementia (Bose and Beal 2016). These clinical findings suggest the enzymatic activity of SIRT1 was reduced in specific brain regions that increase vulnerability to the effect of neurotoxins.

Thus, SIRT1 over-expression was reported to inhibit aggregation of α-synuclein through activation of the PGC-1α signaling pathway and molecular chaperones (Trist et al. 2019). As well, single nucleotide polymorphism (SNP) in the promoter region of SIRT1 was linked with the development of PD patients compared to controls (Zhou et al. 2021).

These observations suggest the neuroprotective role of SIRT1 against the development and progression of PD. However, the underlying mechanisms of SIRT1 against PD need to be elucidated.

## Mechanistic role of SIRT1 in PD

### SIRT and autophagy

The autophagy-lysosomal process is a degradation cellular processes engaged with different cellular process in both normal and diseased cells (Zhang et al. 2022). The normal function of the autophagy-lysosomal process is the transfer of different components including misfolded proteins into the lysosomes for degradation through three chief pathways micro-autophagy, macro-autophagy, and chaperon-mediated autophagy (Hemmati-Dinarvand et al. 2019). Macro-autophagy is responsible for removing damaged organelles like injured mitochondria (mitophagy) by creating phagosomes (Guo et al. 2016; Hussain and Kayani 2020). Genetic variation in PD triggers the development of autophagy dysfunction which leads to the accumulation of misfolded proteins and degeneration of DNs (Trist et al. 2019).

It has been shown that increasing SIRT1 expression by NAD, resveratrol, and caloric restriction inhibits p53 and activates FOXO3 with subsequent activation of autophagy. Activated and functional autophagy is linked with life longevity (Cantó and Auwerx 2009).

SIRT1 has an important role in reducing the development and progression of PD neuropathology through the activation of heat shock factor 1 (HSF-1) and autophagy process which inhibits aggregation of α-synuclein (Lehtonen et al. 2019). Upregulation of autophagy by SIRT1 reduces the accumulation of α-synuclein and misfolded proteins with maintaining cellular homeostasis (Shaikh et al. 2019). A previous study showed that SIRT1 activation inhibits fragmentation and accumulation of prion protein through activation of the autophagy (Wang et al. 2022; Obrador et al. 2021). It has been reported that SIRT1 promotes autophagy process via stimulation expression of tuberous sclerosis 2 proteins and FOXO1 which increase the survival of neuronal tissues (Obrador et al. 2021).

In addition, SIRT1 attenuates the development of apoptosis by inducing autophagy in glucose deprivation–perfusion injury (Ghiasi et al. 2019). It has been shown that SIRT1 activators increase the expression of autophagy markers and decrease deposition of α-synuclein formation and aggregation through inhibition of protein hyperphosphorylation (Gelders et al. 2018). Thus, SIRT1 inhibitors increase the accumulation of α-synuclein through the diminution of the autophagy process (Wang et al. 2021). HSF-1 is an essential molecular chaperon that inhibits the aggregation of the misfolded proteins and α-synuclein. It also protects DNs from the effect of inflammation and oxidative stress (Kim et al. 2018). Therefore, antioxidant melatonin reduces MPTP-induced PD through the upregulation of HSF-1 (Kim et al. 2018). Molecular chaperones are one of most primary post-translation modifications involved in protein synthesis and folding of polypeptides to pass into organelles and the ubiquitin–proteasome system (UPS) for induction of autophagy. This process can decrease the accumulation and cytotoxic effect of misfolded proteins (Sahoo et al. 2022). Molecular chaperones are upregulated by HSF-1 to maintain protein homeostasis (Sahoo et al. 2022). Remarkably, SIRT1 is not co-localized with phosphorylated α-synuclein; therefore, the effect of SIRT1 on phosphorylated α-synuclein is indirect and maybe through activation of HSF-1 and autophagy (Perren et al. 2021). As well, SIRT1 activates autophagy via the expression of AMPK as reported in a previous study (Ghavami et al. 2014; Masi et al. 2019). SIRT1 enhancing autophagy effect plays a critical role in modulation of the activity of DNs with reduction accumulation of misfolded proteins and α-synuclein. Thus, the SIRT1/autophagy axis is essential in prevention of the development and progression of PD neuropathology.

### SIRT1 and oxidative stress

Brain tissue has superior oxygen metabolism with low antioxidant capacity resulting in oxidative stress which damages DNs in the SN (Parga et al. 2021). As well, the development of mitochondrial dysfunction due to genetic mutation and environmental toxins promote the development of oxidative stress and injury of DNs in the SN by the oxidative stress mechanism (Le et al. 2019). Oxidative stress in the early phase of PD triggers a compensatory mechanism by increasing antioxidant enzymes (Kaewmool et al. 2020). In this state, PGC-1α is activated resulting in the activation of expression of the antioxidant enzymes including superoxide dismutase and catalase (Ogawa et al. 2020). PGC-1α is directly activated by SIRT1 which enhances the expression of antioxidant enzymes (Chan et al. 2018). Later on, as DNs damage progress, the antioxidant capacity is reduced with the augmentation of oxidative stress injury (Qi et al. 2018). It has been shown that SIRT1 prevents the development of endothelial dysfunction through attenuation progression of oxidative stress by multiple mechanisms including SIRT1/SOD, SIRT1/FOXO, SIRT1/eNOs, and SIRT1/NF-κB (Kang et al. 2020). In addition, increasing ROS and oxidative stress by the aging process attenuate expression and functional activity of SIRT1 with further progress of oxidative stress and activation of inflammatory signaling pathway NF-κB leading to advanced inflammatory and oxidative stress injury (Bouchez and Devin 2019). This observation suggests a crosstalk between SIRT1 and oxidative stress as well as NF-κB in the aging process.

Oxidative stress is linked with the degeneration of DNs through the induction of excitotoxicity, inflammation, and NO toxicity (Popov 2020). In addition, oxidative stress promotes DNA injury, lipid, and protein oxidations through increasing production of peroxynitrite and hydroxyl free radicals which also cause the degeneration of DNs (Alam et al. 2021). Besides, proteasome dysfunction in PD also triggers the development of oxidative stress with progressive neuronal injury (Nishida et al. 2020). The molecular mechanism for oxidative stress-induced dopaminergic neurotoxicity is not well elucidated.

SIRT1 is regarded as an anti-aging molecule, and SIRT1 activators may play an important role in the mitigation of oxidative stress linked with aging (Liu et al. 2022). Moreover, SIRT1 activators improve mitochondrial function, mitochondrial enzymes like succinate dehydrogenase, and reduce the production of ROS in neuronal cells (Erekat 2018). Besides, SIRT1 activators improve mitochondrial biogenesis and metabolism preventing the development of mitochondrial dysfunction and propagation of oxidative stress (Liu et al. 2018). Of note, SIRT1 improves insulin sensitivity by inhibiting the development of mitochondrial dysfunction and the progression of oxidative stress (Yu et al. 2019).

It has been shown that PD-associated neuroinflammation is mainly controlled by activated microglia and to a lesser extent by oligodendrocytes and astrocytes (Qazi et al. 2021). Interestingly, α-synuclein and other associated proteins like DJ-1, parkin, and LRRK2 play a perilous role in the activation of microglia (Calopa et al. 2010). Markedly, extracellular α-synuclein from damaged DNs activates toll-like receptor 2 (TLR2) leading to microglia activation and release of pro-inflammatory cytokines with the development of neuroinflammation (Blandini et al. 2004). SIRT1 plays a critical role in reducing α-synuclein-induced neurotoxicity. In addition, α-synuclein pathology is linked with bioenergetics dysfunction in PD by inhibiting the expression of SIRT1 and the development of aberrant mitochondrial morphology (Yan et al. 2014). Therefore, SIRT1 activation could be an effective way to reduce α-synuclein accumulation and associated degeneration of DNs.

Microglia activation is linked with the development of sporadic PD neuropathology (Gaki and Papavassiliou 2014). In response to environmental toxins, microglia are extremely activated leading to the release of ROS which through the development of oxidative stress can lead to DNs neurotoxicity and injury (Gaki and Papavassiliou 2014). As well, activated microglia expressing a higher concentration of NADPH oxidase enzyme promotes inflammatory reactions and the release of ROS (Hirsch and Standaert 2021). NADPH oxidase promotes the expression of neuronal angiotensin II which is regarded as a potent inducer of oxidative stress and inflammation (Kouli et al. 2020). Notably, MPTP-induced PD in animal model studies has been reported to be mediated by neuronal angiotensin II (Batiha et al. 2022). Thus, NADPH oxidase and angiotensin II inhibitors could be effective in the management of PD through the mitigation of oxidative stress and neuroinflammation.

In this state, activating SIRT1 may reduce microglia activation and the development of neuroinflammation through its anti-inflammatory effect (He et al. 2016). It has been shown that SIRT1 can inhibit microglia activation-induced neurotoxicity and neurodegeneration (He et al. 2016). Activation of SIRT1 by protocatechuic acid limits microglia activation and associated neuronal cell deaths (Paik et al. 2021). Similarly, pemafibrate, a selective PPARα agonist, inhibits inflammatory reactions through suppression of microglia activation and pro-inflammatory cytokine expression via the SIRT1-dependent pathway (Wang et al. 2019). More selective action of SIRT1, blocking of NADPH oxidase activity reduce the inflammatory reactions and development of endothelial dysfunction in vascular aging (Fan et al. 2020). More robust action of SIRT1 against the development of inflammatory reactions may be through the inhibition of angiotensin II (Jiang and Dickson 2018). Over-expression of SIRT1 can reduce angiotensin II-mediated inflammation and endothelial dysfunction (Qiao et al. 2018).

**Fig. 2:**
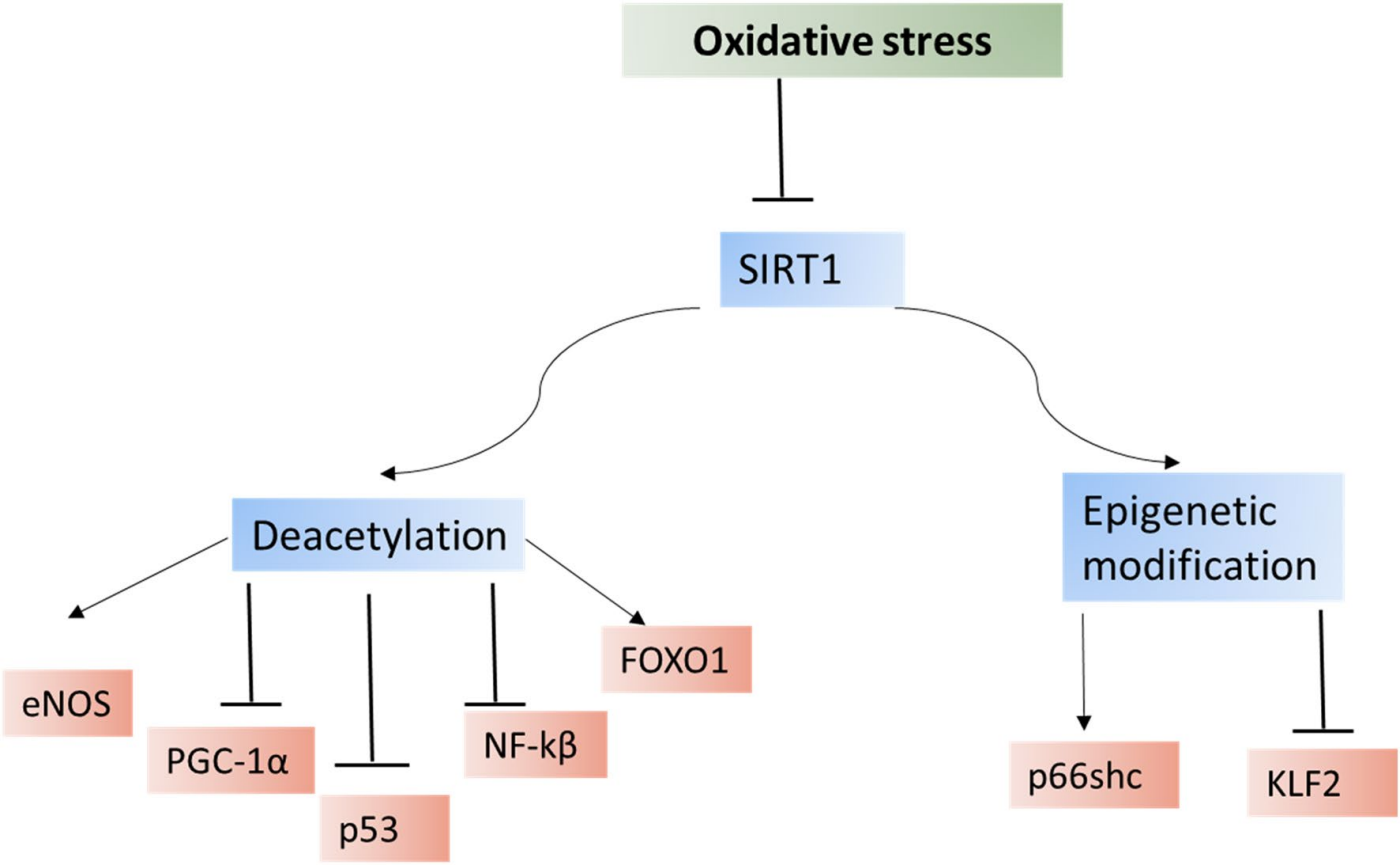
SIRT1 and oxidative stress. Oxidative stress inhibits the expression of SIRT1. SIRT1 deacetylases different targets with activation of the endothelial nitric oxide synthase (eNOS) and peroxisome proliferator-activated receptor gamma co-activator 1 alpha (PGC-1α) with inhibition of p53, forkhead box O1 (FOXO1), and nuclear factor kappa B (NF-κB). SIRT1 via epigenetic effects activates kruppel-like factor 2 (KLF2) and inhibits p66shc gene expression

Taken together, SIRT1 and SIRT1 activators can mitigate PD neuropathology through the modulation of oxidative stress in DNs of the SN (Fig. 2).

### SIRT1 and mitochondrial dysfunction

PD neuropathology is associated with the development of mitochondrial dysfunction which induces the degeneration of DNs in the SN. Under chronic and severe oxidative stress, the mitochondria are subjected to substantial irreversible structural and functional changes in the inner mitochondrial membrane leading to membrane collapse and the development of mitochondrial dysfunction (Qiao et al. 2018). Administration of MPTP or rotenone in mice promotes the development of mitochondrial dysfunction via inhibition of mitochondrial complex I, suggesting that inhibition of mitochondrial complex I and propagation of mitochondrial dysfunction could be the primary event in PD neuropathology. MPTP, rotenone, and other mitochondrial toxins increase the opening of mitochondrial permeability transition and generation of ROS. The catalytic subunit of mitochondrial complex I in the frontal cortex is distorted by ROS in PD due to a defect in the mitochondrial DNA. Defects in mitochondrial complex I induce activation of apoptosis-inducing factors which increase the sensitivity of DNs to the effect of neurotoxins (Boxberger et al. 2019).

Different studies reported that mitochondrial dysfunction via the production of ROS augments the degeneration of DNs in the SN. Therefore, the development of mitochondrial dysfunction due to genetic and environmental factors induces degeneration of DNs through ROS-dependent pathways. Dysregulation of transcription factors like PGC-1α due to exogenous pathological factors leads to the inhibition of mitochondrial biogenesis (Bougea et al. 2019). PGC-1α deregulation is linked with the development and progression of PD through increasing sensitivity of DNs to the effect of neurotoxins like MPTP. Activation of PGC-1α by resveratrol is associated with significant protection of DNs in the SN. Deficiency of PGC-1α promotes clearance of α-synuclein. It has been reported that mutation in the parkin gene in PD induces mitochondrial dysfunction and autophagy abnormality. These changes reduce the functional activity of PGC-1α with subsequent development of PD. However, massive over-expression of PGC-1α is linked with dopamine depletion and degeneration of DNs (Codolo et al. 2013).

These verdicts proposed that mitochondrial dysfunction and defect in mitochondrial biogenesis promote the development and progression of PD neuropathology by inducing the degeneration of DNs. Therefore, targeting mitochondrial dysfunction could be effective in the mitigation of PD pathogenesis (Codolo et al. 2013).

**Fig. 3:**
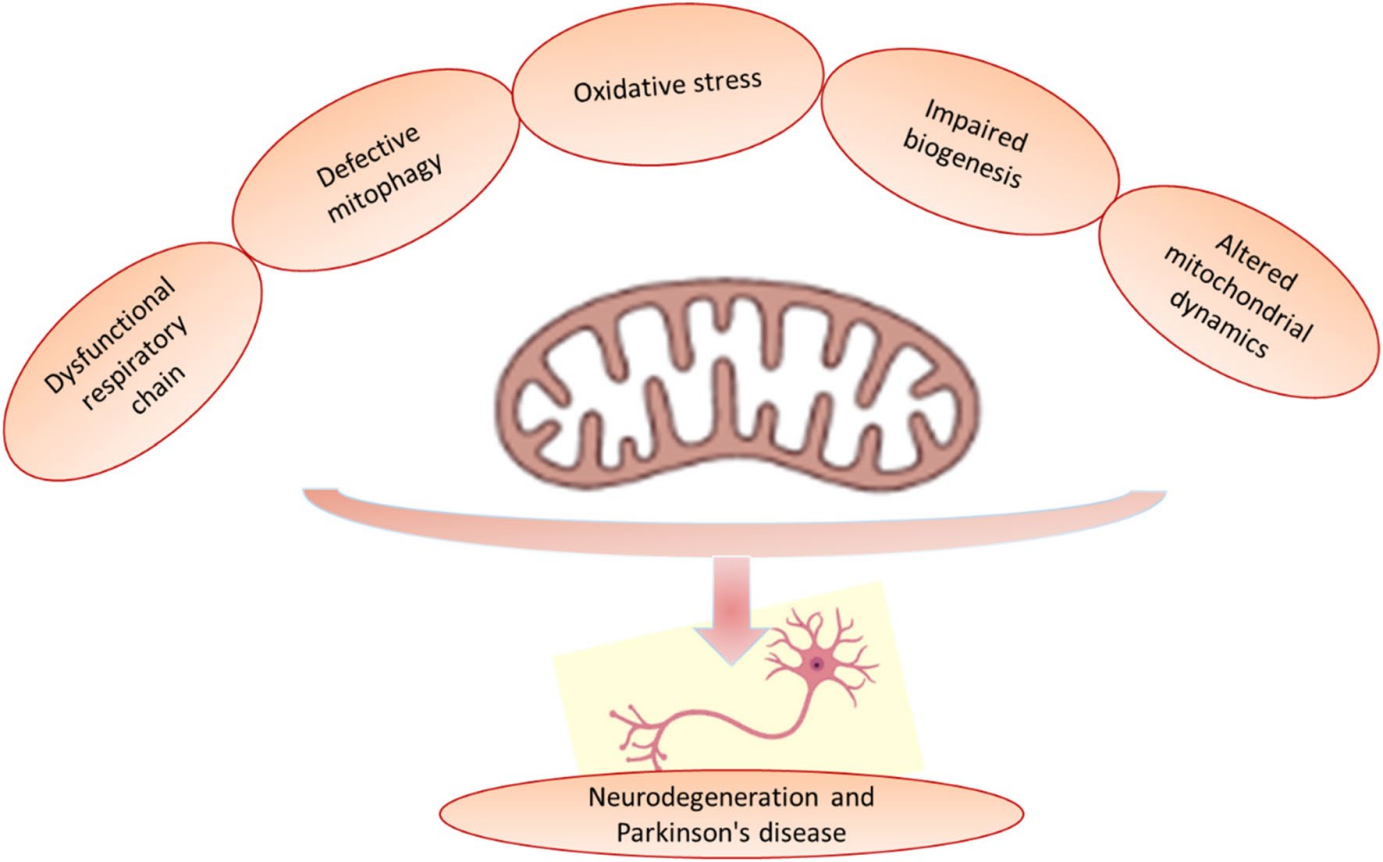
Mitochondrial dysfunction and development of Parkinson’s disease (PD). Calcium imbalance, altered mitochondrial dynamics, impaired biogenesis, oxidative stress, defective mitophagy, impaired mitochondrial trafficking, and dysfunctional respiratory chain lead to development of mitochondrial dysfunction with subsequent progression of PD due to degeneration of the dopaminergic neurons

Mitochondrial biogenesis is involved in the regulation transcription of the mitochondrial and nuclear DNA-encoded genes. Mitochondrial biogenesis is regulated and under the control of PGC-1 effect which regulates the expression of mitochondrial DNA and genes. Deacetylation of PGC-1 by SIRT1 increases its activity (Codolo et al. 2013). Therefore, SIRT1 acts as a redox and metabolic sensor to regulate mitochondrial biogenesis and prevent the development of mitochondrial dysfunction. An experimental study observed that SIRT1 activators improve insulin sensitivity and muscle endurance by increasing mitochondrial biogenesis (Nishida et al. 2020). As well, SIRT1 activation protects DNs from the neurotoxic effect of MPTP by augmentation of mitochondrial biogenesis (Al-Kuraishy et al. 2022). Thus, SIRT1 and SIRT1 activators can reduce the development of mitochondrial dysfunction which is involved in the pathogenesis of PD. Different cellular and pathological disorders including calcium imbalance, altered mitochondrial dynamics, impaired biogenesis, oxidative stress, defective mitophagy, impaired mitochondrial trafficking, and dysfunctional respiratory chain lead to the development of mitochondrial dysfunction with subsequent progression of PD due to degeneration of DNs (Fig. 3).

### SIRT1 and apoptosis

Apoptosis is regarded as a final pathway involved in the loss of DNs in the SN in PD and other degenerative brain diseases (Al-Kuraishy et al. 2022). Multiple signaling pathways including caspase-dependent and caspase-independent pathways mediate nuclear degradation and neuronal apoptosis in the SN (Al-Kuraishy et al. 2022). Activation of initiators like caspase-9 and caspase 8 in the intrinsic or extrinsic pathways, respectively, promotes the activation of executioner caspases and pro-apoptotic pathways causing DNA injury and apoptosis (Calopa et al. 2010). A case–control study involving 89 PD patients (56 treated and 33 untreated) compared to 33 healthy controls showed that circulating CD4-expressing Fas, a biomarker of apoptosis, was increased compared to controls. Likewise, there was a negative correlation between Bcl-2 serum level and PD severity in patients treated with dopaminergic agonists (Calopa et al. 2010).

**Fig. 4:**
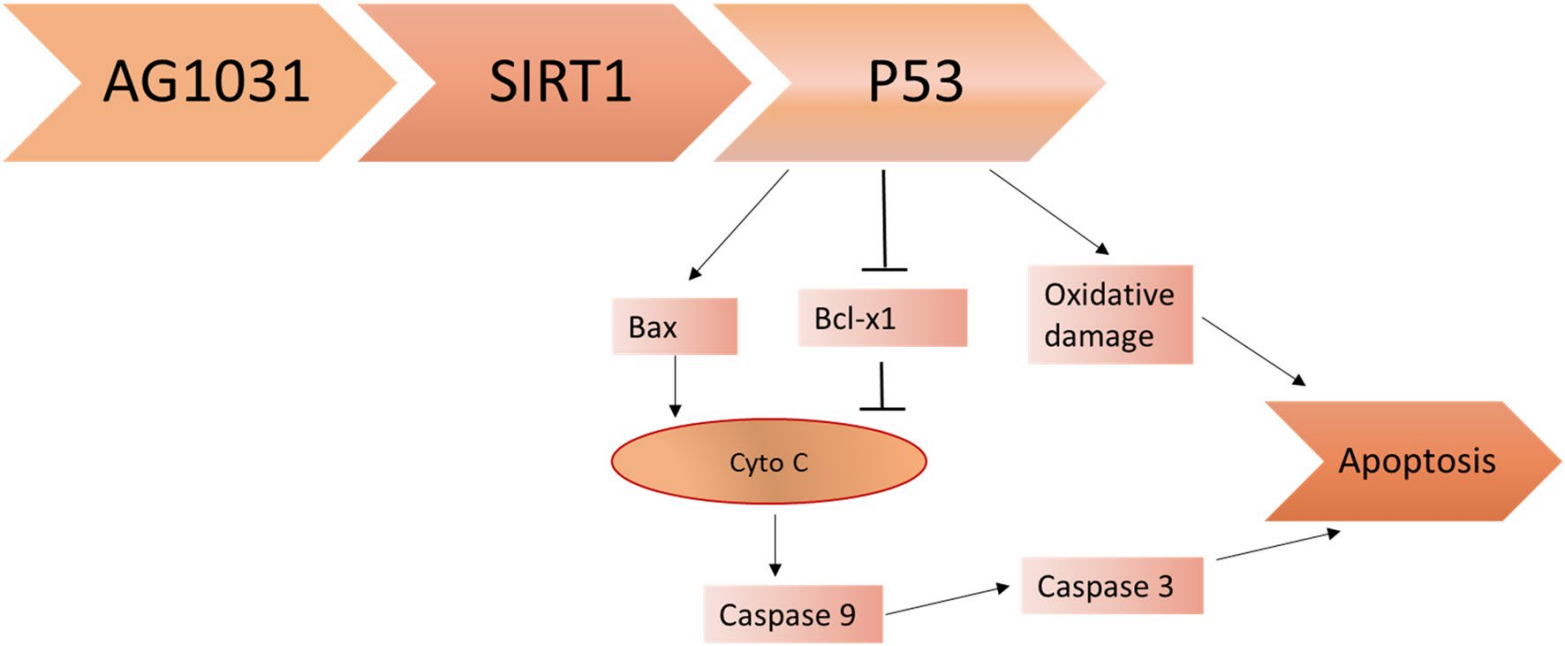
SIRT1 inhibitor, AG1031, and development of apoptosis. SIRT1 inhibits the expression of p53 as well as the development and progression of apoptosis. The SIRT1 inhibitor, AG1031, promotes apoptosis through uncontrolled p53 activation. p53 induces apoptosis directly by triggering the development of the oxidative stress or indirectly through activation of the pro-apoptotic Bax gene which stimulates cytochrome c (cyto c) and caspase-9 with subsequent activation of caspase-3 leading to apoptosis

It has been shown that SIRT1 and SIRT1 activators reduce the risk of PD in MPTP-model mice through the suppression of pro-apoptotic and apoptotic pathways (Calopa et al. 2010). During the development of apoptosis, p53 is released causing activation expression of anti-apoptotic proteins. Yu et al. (Singh et al. 2020) demonstrated that SIRT1 attenuates apoptosis both in vitro and in vivo through the activation of microRNA-494 in the spinal cord injury model. Besides, inhibition of SIRT1 by miR-132 promotes degeneration of DNs in the SN by induction of apoptotic pathway through a p53-dependent pathway (Hunot et al. 1997). These findings proposed that SIRT1 inhibits the development and progression of PD neuropathology by attenuating the apoptotic process. In sum, p53 induces apoptosis directly by triggering the development of oxidative stress or indirectly through activation of the pro-apoptotic Bax gene which stimulates cytochrome c (cyto c) and caspase-9 with subsequent activation of caspase 3 leading to apoptosis. SIRT1 inhibits the expression of p53 and the development and progression of apoptosis. SIRT1 inhibitor, AG1031, promotes apoptosis through uncontrolled p53 activation (Hunot et al. 1997) (Fig. 4).

### SIRT1 and inflammatory signaling pathways

Notoriously, PD neuropathology is linked with the inflammatory changes and the release of pro-inflammatory cytokines due to the activation of the inflammatory signaling pathways (Ghosh et al. 2007). DNs in the SN are liable to inflammatory attack due to the accumulation of the inflammatory proteases and cytokines with the succeeding commencement of DNs degeneration (Flood et al. 2011; Dolatshahi et al. 2021). Microglia activation in the SN due to oxidative stress activates apoptosis of DNs by the release of pro-inflammatory cytokines (Buddhala et al. 2015). As well, activation of NADPH oxidase and development of oxidative stress in PD also participate in the activation of inflammatory signaling pathways, the release of pro-inflammatory cytokines, and development of neuroinflammation (Buddhala et al. 2015). Genetic and epidemiological studies support the complicated role of neuroinflammation in PD pathophysiology (Xiang-Sheng et al. 2021). Activated microglia which correlated with α-synuclein was increased in the amygdale of PD patients compared to the control (Shi et al. 2011). The CD4 T cells and activated microglia are interrelated in the initiation of neuroinflammation and degeneration of DNs (Shi et al. 2011). Though, the selective effect of inflammatory signaling pathways in PD pathophysiology needs to be clarified more by preclinical and clinical studies.

#### NLRP3

Nod-like receptor pyrin 3 (NLRP3) inflammasome is the nucleotide-binding domain and leucine-rich repeat-containing family and the pyrin family can form a multiprotein complex (Park et al. 2014). The main function of NLRP3 inflammasome is the activation of caspase-1 and maturation of IL-1β and IL-18 (Park et al. 2014). NLRP3 inflammasome is triggered by diverse stimuli including alternative and non-canonical pathways (Vellimana et al. 2018). NLRP3 inflammasome is activated by NF-κB and sphingosine-1 phosphate (Hattori et al. 2014). NLRP3 inflammasome is involved in the pathogenesis of PD (Cantó and Auwerx 2009). NLRP3 inflammasome induces the release of the pro-inflammatory cytokines and the progress of neuroinflammation and degeneration of DNs by induction of pyroptosis (Cantó and Auwerx 2009; Wang et al. 2022). Additionally, accumulation of the α-synuclein activates stimulation of the microglia with subsequent expression of NLRP3 inflammasome in the SN (Cantó and Auwerx 2009; Wang et al. 2022). Furthermore, systemic activation of NLRP3 inflammasome encourages the accumulation of α-synuclein and degeneration of DNs in the SN (Fan et al. 2020). A case–control study that included 67 PD patients compared to 24 healthy controls showed that plasma levels of α-synuclein, NLRP3 inflammasome, caspase-1, and IL-1β were increased in PD patients compared to healthy controls (Fan et al. 2020). Consequently, α-synuclein, NLRP3 inflammasome, and IL-1β plasma levels could serve as biomarkers to monitor PD severity and progression.

Frequent studies demonstrate that higher levels of pro-inflammatory cytokines in the CSF and plasma support the interaction between the brain and the immune system with the development of neuroinflammation and degeneration of DNs in PD (Kang et al. 2021; Chen et al. 2022). IL-1β plasma level, the main component of NLRP3 inflammasome, is increased in PD patients (Qian et al. 2018). These annotations proposed that systemic inflammation via induction of neuroinflammation may lead to the degeneration of DNs and the development of PD. Moreover; increase in α-synuclein plasma level, which is a chief constituent of Lewy bodies, had been reported to be increased in PD patients compared to the healthy controls (Guerreiro et al. 2020). In sequence, α-synuclein can trigger NLRP3 inflammasome with subsequent release of IL-1β with the development of systemic inflammation and neuroinflammation (Zhang et al. 2020).

SIRT1 has an anti-inflammatory effect by inhibiting NLRP3 inflammasome activation in LPS-induced endometritis (Al-Kuraishy et al. 2018). An experimental study illustrated that dioscin attenuates the severity of subarachnoid hemorrhage through inhibition of NLRP3 inflammasome via a SIRT1-dependent pathway. Different studies revealed that SIRT1 has a protective effect against neurodegeneration and neuronal injury (Di et al. 2022). SIRT1 activation by resveratrol leads to a protective effect against complications in cerebral ischemia. As well, over-expression of SIRT1 in mice preserves cerebral blood flow and prevents ischemic–reperfusion injury through modulation of FOXO1, NF-κB, and expression of TLR4. SIRT1 can reduce cerebral vasospasm via modulation of the expression and activation of NLRP3 inflammasome as well as regulation of eNOS. As well, SIRT1 activation promotes ischemic tolerance by increasing HIF-1 through the SIRT1-dependent pathway (Di et al. 2022). These findings proposed that SIRT1 activation represents a promising strategy to reduce ischemic brain injury. Moreover, zafirlukast attenuates the activation of NLRP3 inflammasome via the SIRT1-dependent pathway in hepatocytes. Likewise, nicotinic acid reduces vascular inflammation through inhibition of NLRP3 inflammasome signaling pathway (Al-Kuraishy et al. 2021). These observations suggest that SIRT1, through inhibition of the NLRP3 inflammasome, can mitigate PD neuropathology. SIRT1 activation by arginine (Arg) inhibits generation of the ROS-mediated NLRP3 inflammasome activation. SIRT1 directly inhibits NLRP3 inflammasome activation and conversion of pro-caspase to caspase-1 which converts pro-IL-1β to IL-1β (Al-Kuraishy et al. 2021).

#### NF-κB

**Fig. 5:**
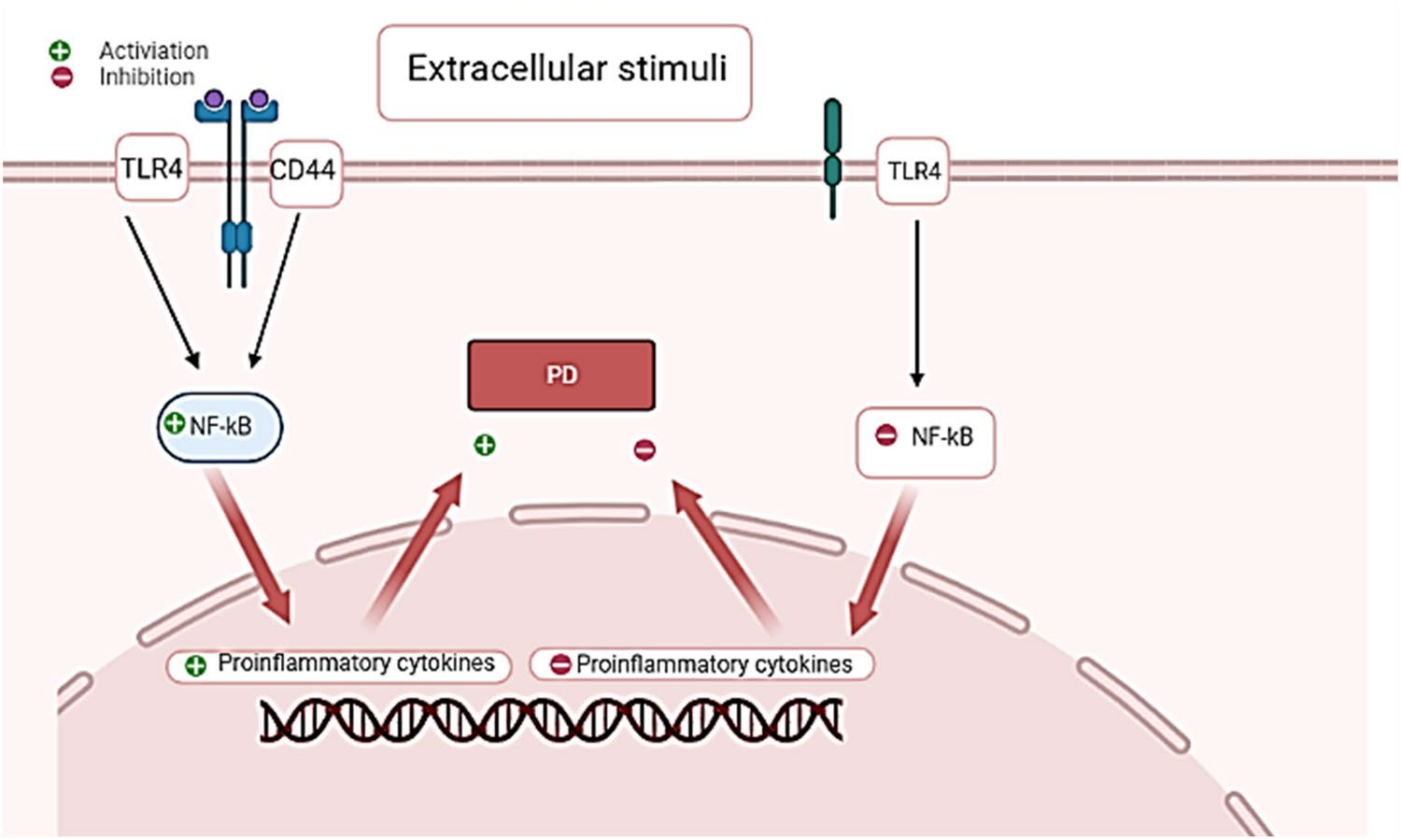
Role of NF-κB in the development of PD. Extracellular stimuli through activation of TLR4 in the presence of CD44 promote NF-κB activation and the release of pro-inflammatory cytokines with development of PD. However, deficiency of CD44 attenuates NF-κB activation and the development of neuroinflammation in PD

NF-κB is a DNA-binding protein prerequisite for the transcription of pro-inflammatory cytokines and chemokines. NF-κB is under the control of extracellular stimuli, it is inhibited by an inhibitor of κB (IκB) which sequesters NF-κB in the cytosol and averts its localization (Al-Kuraishy et al. 2016). Nevertheless, cytokines inhibit IκB with succeeding activation of NF-κB and promulgation of inflammatory disorders. NF-κB is also concerned with the pathogenesis of PD via the induction of inflammation-mediated degeneration of DNs in the SN (Herath et al. 2016). Extracellular stimuli through activation of TLR4 in presence of CD44 promote NF-κB activation and release of pro-inflammatory cytokines with the development of PD. However, deficiency of CD44 attenuates NF-κB activation and the development of neuroinflammation in PD (Fig. 5).

Immune dysregulation by aging promotes the activation of NF-κB with succeeding neuronal injury and neuroinflammation with the development of PD (Shi et al. 2016). Results from post-mortem studies advocate the role of NF-κB in the degeneration of DNs in the SN. Activation of NF-κB with induction of neuronal apoptosis was recognized in PD patients compared to the controls (Shi et al. 2016). Thus, selective inhibition of NF-κB prevents degeneration of DNs in the SN in the mouse model of PD (Imfeld et al. 2012). Likewise, targeting of NF-κB pathway in a mouse model of PD may prevent the progression of PD (Moore et al. 2013a). Different drugs and herbals like pioglitazone, salmeterol, and curcumin delay the degeneration of DNs in the SN by inhibiting NF-κB which is concerned with the progression of neuroinflammation and injury of DNs (Dolatshahi et al. 2021). An up-to-date finding demonstrated that α-synuclein released from injured DNs triggers activation of NF-κB and release of pro-inflammatory cytokines with further aggravation of DNs in a positive-loop fashion (Dolatshahi et al. 2021). These conclusions proposed that NF-κB could be a therapeutic target in the management of PD. Particularly, the Aβ_1-42_ level in the CSF is reduced and not correlated with motor dysfunction in PD patients compared to the controls (Venna et al. 2014; Chen et al. 2016). Nonetheless, Aβ_1-42_ inhibits BBB P-glycoprotein through induction of NF-κB with further reduction in clearance of Aβ_1-42_ (Patil et al. 2014). For this reason, NF-κB not only induces DNs in the SN but also increases the PD severity through the accumulation of Aβ_1-42_ and α-synuclein.

**Fig. 6:**
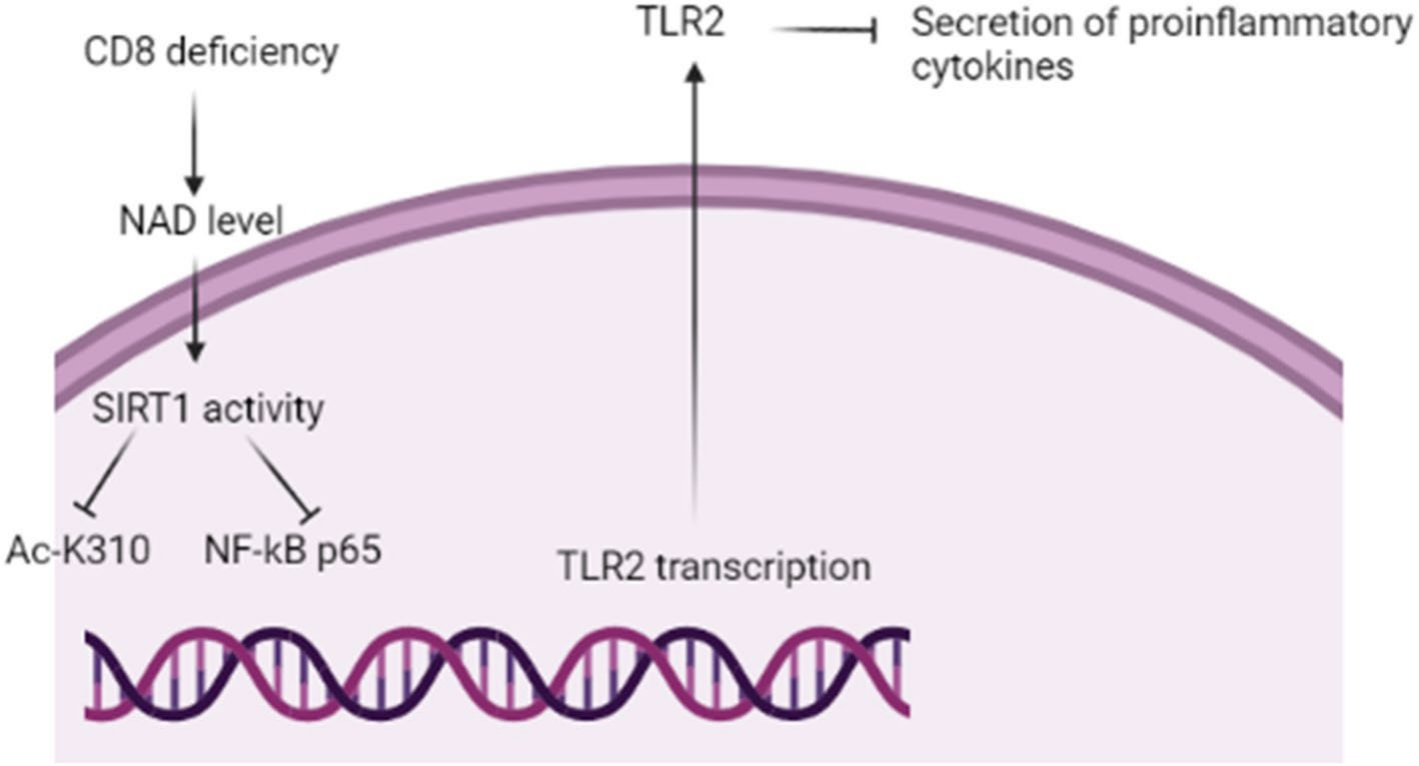
CD38 and SIRT1 pathway. CD38 deficiency promotes NAD and SIRT1 activation which inhibits the expression of Ac-K310 and NF-κB with subsequent inhibition of the release of the pro-inflammatory cytokines

Taken together, NF-κB and NLRP3 inflammasome are activated in PD neuropathology and linked with the development of neuroinflammation (Paudel et al. 2020). It has been shown that antagonistic crosstalk between SIRT1 and NF-κB is present to regulate energy metabolism and innate immunity (Liao et al. 2021). NF-κB regulates immunity defense while SIRT1 controls inflammatory signaling pathways and cellular survival. Notably, NF-κB activates glycolytic energy during inflammation while SIRT1 regulates inflammation and oxidative stress. Energy balance and inflammation are highly dysregulated in PD. In addition, the SIRT1 factor inhibits NF-κB-mediated immune activation and response (Cao et al. 2022). It was revealed that cannabidiol improves autophagy and mitochondrial dysfunction via activation of SIRT1 and inhibition of the NF-κB signaling pathway. It was observed that berberine attenuates LPS-induced cognitive dysfunction through regulation of the SIRT1/NF-κB axis in mice (Docrat et al. 2020). Interestingly, CD38 deficiency promotes NAD and SIRT1 activation which inhibits the expression of Ac-K310 and NF-κB with subsequent inhibition release of pro-inflammatory cytokines. CD38 expression is correlated with aging with an increased risk for the development of neurodegenerative brain diseases and neuroinflammation (Gelders et al. 2018). CD38 reduces the neuroprotective NAD with subsequent attenuation of anti-inflammatory SIRT1 and increment of inflammatory signaling NF-κB leading to the progression of neuroinflammation and the development of neurodegenerative brain diseases (Fig. 6). Therefore, inhibition of NF-κB by SIRT1 may attenuate neuroinflammation and the development of PD.

In PD, only a few molecular mechanisms have been clarified so far in the neurodegenerative cascade. In such a multifaceted picture, it is particularly important to identify experimental models that simplify the study of the different networks of proteins/genes involved. Cellular models that reproduce some of the features of the neurons that degenerate in PD have contributed to many advances in our comprehension of the pathogenic flow of the disease (Gelders et al. 2018). In particular, the pivotal biochemical pathways (i.e., apoptosis and oxidative stress, mitochondrial impairment, dysfunctional mitophagy, unfolded protein stress, and improper removal of misfolded proteins) have been widely explored in cell lines, challenged with toxic insults or genetically modified. The central role of α-synuclein has generated many models aiming to elucidate its contribution to the dysregulation of various cellular processes. However, classical cellular models appear to be the correct choice for preliminary studies on the molecular action of new drugs or potential toxins and for understanding the role of single genetic factors. In addition, the availability of novel cellular systems, such as cybrids or induced pluripotent stem cells, offers the chance to exploit the advantages of an in vitro investigation, although mirroring more closely the cell population being affected (Wang et al. 2021). Accordingly, PD neuropathology is multifactorial and related to genetic and epigenetic factors. Therefore, the definitive single cause of PD is not simple as different cellular processes are intricate together in the pathogenesis of PD. SIRT1 affects PD neuropathology at different molecular and cellular levels, though it’s main effect remains not fully elucidated (Cuyàs et al. 2018). In this bargain, preclinical molecular studies are recommended in this regard to clarify the chief mechanistic pathway involved in SIRT1 effect on PD progression.

## Role of SIRT1 activators in PD

SIRT1 activators of different types can mitigate the progression of PD neuropathology through the regulation of oxidative stress, inflammatory disorders, mitochondrial dysfunction, apoptosis, and inflammatory signaling pathways (Cuyàs et al. 2018). However, each SIRT1 activator has a specific mechanistic pathway for attenuating the pathogenesis of PD (Table 1).

**Table 1:** SIRT1 activators of different types can mitigate the progression of PD neuropathology

Study type	Findings	Ref
Experimental study	Metformin 20 mg/kg had a neuroprotective effective through epigenomic and integrated stress responses in diabetic mice via activation of SIRT1	Docrat et al. (2020)
Experimental study	Metformin 100 mg/kg has led to attenuation of the degeneration of DNs in the nigrostriatal region through activation of SIRT1/PGC-1α pathway	Bayliss et al. (2016)
Experimental study	SIRT1 activators metformin and resveratrol improved the mitochondrial function	Price et al. (2012)
In silico study	Metformin is a direct SIRT1 activator which prevented aging-associated disorders when the activity of NAD and SIRT1 is decreased	Cuyàs et al. (2018)
Experimental study	Metformin 100 mg/kg reduced the development of the oxidative stress and regulated the autophagy in rats with high-fat diet through modulation of SIRT1	Ren et al. (2020)
A systematic review	Resveratrol had a neuroprotective effect against PD development through different mechanisms including anti-inflammatory, antioxidant, and anti-apoptotic effects with SIRT1 expression	Dos Santos et al. (2022)
A randomized, double-blind study	Resveratrol improves hippocampal volume and neurogenesis in PD patients	Köbe et al. (2017)
Review study	Low dose of resveratrol promoted the SIRT1 expression while high dose promoted the AMPK activation	Singh et al. (2019)
Experimental study	Lamin A protein is regarded as a potent SIRT1 agonist that mediated resveratrol-induced SIRT1 expression in mice	Ghosh et al. (2013)
Experimental study	Reduction of the expression of lamin A protein promoted the degeneration of DNs in midbrain with increasing DNs loss and the development of PD	Collier et al. (2017)
In vitro study	SRT-1720 was effective in AD through modulation of the mitochondrial autophagy and biogenesis via the SIRT1-dependent pathway	Ye et al. (2021)

### Metformin

Metformin is an insulin-sensitizing drug used as first-line therapy in the management of type II diabetes mellitus (T2DM). As well, it is indicated in the management of polycystic ovary, obesity, and insulin resistance (IR) (Singh et al. 2019). Metformin can cross BBB and accumulate in some brain regions affecting various CNS functions by inhibiting neuronal apoptosis with promoting the neurogenesis process by activation of neuronal protein kinase C (PKC). In addition, metformin promotes the synaptogenesis by increasing the expression of synaptic-related protein and neurotrophic factors. Similarly, metformin attenuates glutamatergic dysfunction which is linked with the development of neurodegenerative brain diseases as in AD and PD (Cao et al. 2007).

Metformin has a potential neuroprotective effect against the progress of degenerative brain diseases like AD and PD. Metformin decreases AD risk in T2DM patients as recognized by cross-sectional and longitudinal analysis (Al-Kuraishy et al. 2022). Metformin therapy for more than 4 years reduces the risk of the development of degenerative brain diseases (Liu and Zhou 2013). In contrast, numerous studies exposed that long-term metformin therapy was associated with an increasing risk for the development of cognitive impairment and AD (Ghosh et al. 2013). The neuroprotective effect of metformin is mostly mediated by AMPK which attenuates aggregation of Aβ and tau protein hyperphosphorylation. Furthermore, metformin advances neurogenesis, angiogenesis, synaptic plasticity, and induction of autophagy (Maynard et al. 2022). Metformin enhances neuroprotective brain-derived neurotrophic factor (BDNF) leading to the inhibition of neuroinflammation and oxidative stress-induced neuronal injury. Metformin exerted a neuroprotective effect through the inhibition of apoptosis and activation of autophagy in mice with experimental spinal cord injury (Sarge and Park-Sarge 2009). An experimental study demonstrated that the administration of metformin 500 mg/kg in mice orally for 21 days reduced oxidative stress levels and locomotor severity in MPTP-induced PD (Patil et al. 2014). It was demonstrated that metformin led to a protective effect against the development of PD by different mechanisms including inhibition generation of α-synuclein, production of oxidative stress, attenuation of mitochondrial dysfunction, and modulation of the autophagy process through the AMPK-dependent pathway.

In a large cohort of T2DM patients, establish a 2.2-fold increased risk to develop PD in T2DM patients. Entertainingly, the treatment with sulfonylurea, another anti-hyperglycemic agent, considerably increased the risk of PD but it was alleviated for those patients who received a co-therapy with metformin. Though, the administration of metformin alone did not prove to avert the development of PD in diabetic patients (Brakedal et al. 2017). Other clinical studies showed a positive correlation between metformin therapy and PD. On the contrary, a longitudinal study analyzed a 5-year follow-up in more than 5500 veterans with T2DM patients revealed that metformin therapy for more than 4 years notably decreased the risk of developing PD (Shi et al. 2019). A systematic review and meta-analysis showed a lack of correlation between metformin therapy and PD development (Qin et al. 2021). These conflicting results may derive from a high degree of heterogeneity among clinical studies, in terms of population, treatment regime, follow-up lengths, and adjusted factors. In these studies, the average age of the enrolled subjects was 60–65 years, which corresponds to the mean age of onset of PD, although the incidence of PD increases with age (1–2% over 65 years old and more than 5% over 80 years old). However, the follow-up usually took place for an additional 6 years on average, possibly underestimating the correlation between metformin consumption and PD (Qin et al. 2021).

As well, metformin has a profound effect on the alleviation of dopaminergic neurodegeneration in the SN and associated neuroinflammation (Shukla et al. 2016). Therefore, metformin could be an effective treatment strategy that may overcome the limitation of PD treatment. These verdicts suggest that metformin has a neuroprotective effect against degenerative brain diseases including PD through modulation of the oxidative stress and neuroinflammation.

Regarding the potential effects of metformin on SIRT1, different preclinical and clinical observed that metformin mediates its neuroprotective and anti-inflammatory effects through activation of the SIRT1 pathway. It was revealed that metformin led to attenuation degeneration of DNs in the nigrostriatal region independent of AMPK activation in mice. The neuroprotective effect in this study was through activation of the SIRT1/PGC-1α pathway. PGC-1α-deficient mice were more susceptible to the neurotoxic effect of MPTP (Ye and Wu 2021). In addition, metformin-induced AMPK activation promotes activation of the SIRT1/PGC-1α pathway suggesting interplay between AMPK and SIRT1/PGC-1α in the neuroprotective effect of metformin. It has been suggested that metformin is regarded as a potent SIRT1 agonist and can interact with the NAD binding site and C-terminal regulatory region of SIRT1 with subsequent activation of the catalytic site of SIRT1. Therefore, metformin regulates SIRT1 activity and prevents aging-associated disorders when the activities of NAD and SIRT1 are decreased (Wahlqvist et al. 2012). Metformin plays a role in the inhibition of hyperglycemia-induced endothelial senescence and the development of endothelial dysfunction. Likewise, metformin reduces the development of oxidative stress and regulates autophagy in rats with high-fat diet through modulation of AMPK, SIRT1, and FOXO1 (Brakedal et al. 2017). Therefore, metformin activates SIRT1 directly or indirectly through the modulation of the oxidative stress and autophagy.

**Fig. 7:**
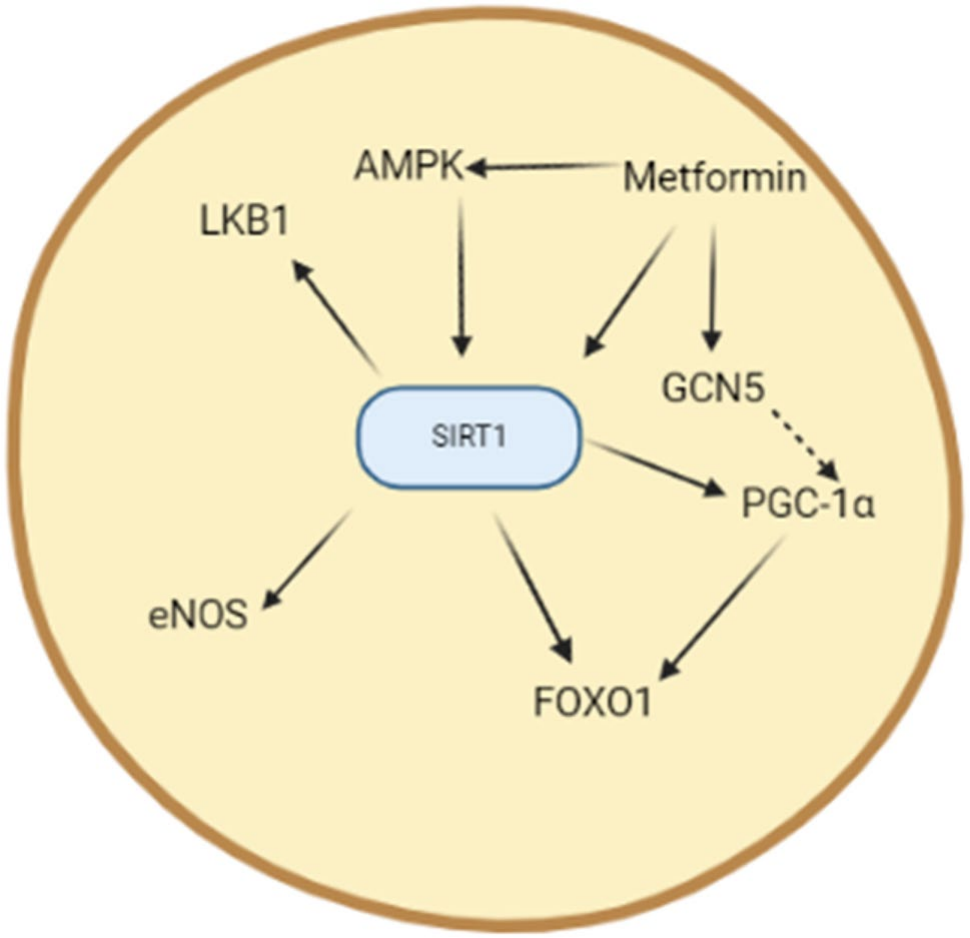
Role of metformin in activation of SIRT1

Collectively, metformin via AMPK, SIRT1, and FOXO1 as well as PGC-1α activation can protect DNs and decrease the PD risk (Fig. 7).

### Resveratrol

Resveratrol is a polyphenol that presents different dietary products; however, its bioavailability is low due to the oxidation of resveratrol by digestive enzymes. Therefore, glycosylation of resveratrol reduces enzymatic degradation and preserver resveratrol bioavailability. Resveratrol through activation of SIRT1 improves cognitive function and reduces AD risk (Brakedal et al. 2017). Numerous studies have shown that resveratrol has anti-inflammatory, antioxidant, and neuroprotective effects against the development and progression of degenerative brain diseases through modulation of hippocampal neurogenesis and accumulation of misfolded proteins. Resveratrol-dependent SIRT1 activation reduces p53 activation and microglial activation. Resveratrol enhances brain function and reduces age-related cognitive dysfunction through the SIRT1 activation mechanism which improves cerebral blood flow and synaptic plasticity. Of note, resveratrol attenuates sevoflurane-induced cognitive decline via enhancement of SIRT1 pathway and inhibition of NF-κB in neonatal mice (Rhee et al. 2020). It was observed that resveratrol was effective in reducing AD pathogenesis in diabetic rats through activation of the SIRT1 pathway. Resveratrol accelerates SIRT1 expression and attenuates memory impairment in diabetic rats. In addition, resveratrol prevents fluoride-induced neurodevelopmental injury through the improvement of mitochondrial biogenesis via the SIRT1 pathway (Moore et al. 2013b). These preclinical studies support the neuroprotective effect of resveratrol against the development of degenerative brain diseases including PD.

**Fig. 8:**
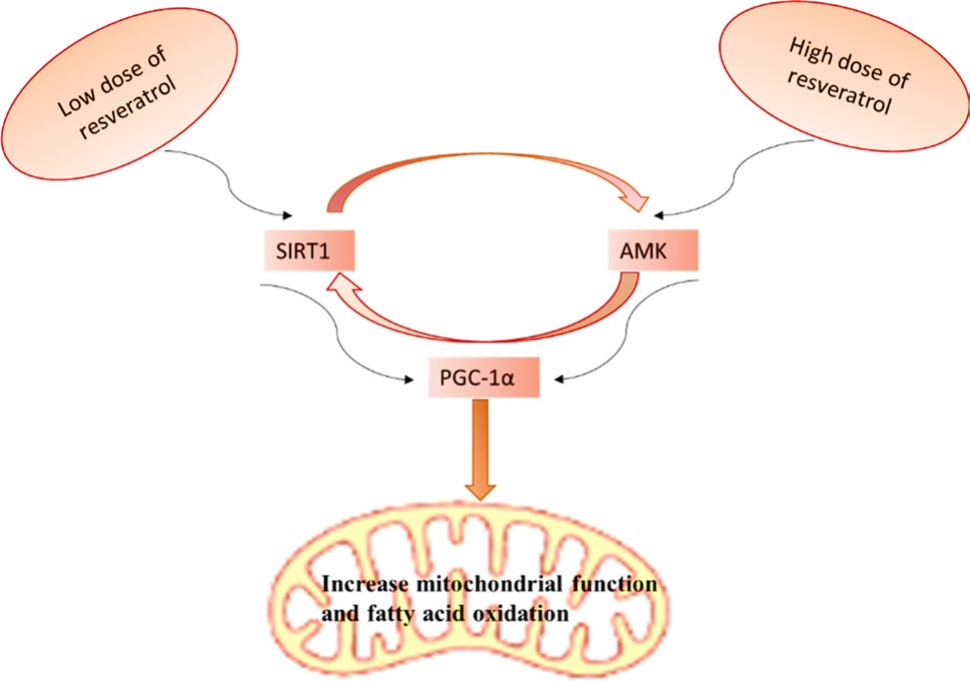
Effects of resveratrol on SIRT1 and AMPK expression

A randomized, double-blind study involving 40 patients with mild cognitive impairment treated with resveratrol 200 mg/day for 26 weeks illustrated that resveratrol improves the hippocampal volume and neurogenesis as documented by imaging findings (Köbe et al. 2017). As well, resveratrol improves memory performance and verbal episodic function through modulation of hippocampal functional connectivity in the older adults. Thus, caloric restriction and resveratrol treatment can reduce brain aging by increasing SIRT1 expression. A systematic review showed that resveratrol has neuroprotective effect against PD development through different mechanisms including anti-inflammatory, antioxidant, and anti-apoptotic effects with SIRT1 expression and subsequent amelioration of neuroinflammation. The molecular effect of resveratrol besides SIRT1 expression is activation of PGC-1α, AMPK, and neural growth factors with inhibition of COX-2, adhesion molecules, and inflammatory signaling pathways. Of interest, a low dose of resveratrol promotes SIRT1 expression while a high dose promotes AMPK activation (Santos et al. 2022). Together, these observations proposed that resveratrol could be effective in the management of PD through modulation of the SIRT1 pathway (Fig. 8).

### Lamin A protein

Lamin A protein also called nuclear lamin is a structural and functional protein involved in the regulation of the cell nucleus. It regulates cell mitosis and apoptosis through the regulation function of the endoplasmic reticulum and caspase activity. Mutations in the expression of lamin A protein induce a series of disorders called laminopathies including premature aging syndrome, cardiomyopathy, muscular dystrophy, and neuropathy. Evidence from in vitro and in vivo findings illustrated that lamin A protein is necessary for SIRT1 expression and activation (Nadwa et al. 2022). Lamin A protein is regarded as a potent SIRT1 agonist that mediates resveratrol-induced SIRT1 expression. It was (Nadwa et al. 2022) demonstrated that SIRT1 activation by resveratrol was mediated and dependent on the activity of lamin A protein. Dysregulation of lamin A protein attenuates PGC1-α/NAD with subsequent reduction of SIRT1 activity and development of mitochondrial dysfunction (Alomair et al. 2022). These verdicts indicated that lamin A protein plays a crucial role in SIRT1 expression and activation.

Lamin protein is the only nuclear protein expressed in the CNS, essential for neuronal differentiation and survival. Lamin A protein is critical for lifespan through inhibition of neurodegeneration (Alomair et al. 2022). It has been reported that mutation with reduced expression of lamin A protein was linked with the development of aging-related degenerative brain disease including PD. Aging-mediated reduction expression of lamin A protein promotes degeneration of DNs in the midbrain with increasing DNs loss and development of PD. Notably, there is an overlap in cellular and molecular alterations of lamin A protein in both PD and Hutchinson-Gilford Progeria syndrome which develops due to a deficiency of lamin A protein (Al-Kuraishy et al. 2022). It has been shown that a reduction in the activity of neuronal lamin A protein is associated with motor circuit disturbances due to a defect in DN neurotransmission. This may explain motor dysfunction associated with aging due to a reduction in the functional activity of lamin A protein. Therefore, age-mediated reduction in lamin A protein activity could be the main cause for the reduction of SIRT1 activity with subsequent development of degenerative brain diseases like PD (Al-Kuraishy et al. 2022). Despite this significant importance regarding the role of lamin A protein, little is known regarding lamin A protein analogs or agonists. Thus, the potential role of lamin A protein agonists need to be clarified in future studies.

### SRT-1720

SRT-1720 is an experimental study used as a small molecule activator of SIRT1 used in research to improve mitochondrial function, though its efficacy and safety in humans were not established. SRT-1720 increases the lifespan of obese mice by improving SIRT1 activity. SRT-1720 is not under clinical improvement through a related compound SRT-2104 pass Phase I clinical trial for the management of metabolic disorders (Al-kuraishy et al. 2022; Dai et al. 2010). In vitro study observed that SRT-1720 was not toxic to cell lines. Recently, SRT-1720 was effective in AD through the modulation of mitochondrial autophagy and biogenesis via the SIRT1-dependent pathway (Shukla et al. 2016; Batiha et al. 2022). Therefore, SRT-1720, through activation of SIRT1, could be a promising future therapy in the management of PD.

Taken together, SIRT1 and SIRT1 activators play a crucial role in the mitigation of PD neuropathology through the amelioration of oxidative stress, inflammatory disorders, mitochondrial dysfunction, apoptosis, and inflammatory signaling pathways.

## Conclusion

SIRT has distinctive enzymatic activities, physiological functions, and sub-cellular localization to control cell-cycle progression, gene expression, and DNA stability by targeting histone and non-histone proteins. SIRT1 enhances synaptic formation and synaptic activity, and therefore can reduce the progression of various degenerative brain diseases like AD and PD. SIRT1 activity is decreased by aging with subsequent increased risk for the development of degenerative brain diseases. Inhibition of SIRT1 promotes inflammatory reactions since SIRT1 inhibits transcription of NF-κB which also inhibits SIRT1 activation via activation of microRNA and miR-34a which reduce NAD synthesis. SIRT1 has antioxidant, anti-inflammatory, and genomic stability effects, and it is highly expressed in microglia and neurons in the human brain; thus, SIRT1 may reduce PD neuropathology. SIRT1 has neuroprotective effects through modulation of the fate precursor cells, inhibition of p53, and attenuation of axonal degeneration. Therefore, downregulation of SIRT1 during aging promotes p53 expression and may increase the vulnerability of neuronal cell deaths. PD neuropathology is linked with the sequence of inflammatory changes and the release of pro-inflammatory cytokines due to the activation of inflammatory signaling pathways. In addition, oxidative stress, inflammatory disorders, mitochondrial dysfunction, and apoptosis contribute mutually to PD neuropathology. Taken together, SIRT1 activators play a crucial role in the mitigation of PD neuropathology through the amelioration of oxidative stress, inflammatory disorders, mitochondrial dysfunction, apoptosis, and inflammatory signaling pathways. Herein, preclinical and clinical studies are prerequisites in this regard.

## Data Availability

All data are available in the manuscript.
